# Evaluation of Turner Syndrome Knowledge among Physicians and Parents

**DOI:** 10.4274/jcrpe.galenos.2019.2019.0041

**Published:** 2020-03-19

**Authors:** Berna Eroğlu Filibeli, Nesrin Havare, Huriye Erbak Yılmaz, Jülide Gülizar Yıldırım, Gönül Çatlı, Bumin N. Dündar

**Affiliations:** 1 University of Health Sciences Turkey, Tepecik Training and Research Hospital, Clinic of Pediatric Endocrinology, İzmir, Turkey; 2 University of Health Sciences Turkey, Tepecik Training and Research Hospital, Clinic of Pediatrics, İzmir, Turkey; 3 Katip Çelebi University, Atatürk Traning and Research Hospital, Clinic of Biochemistry, İzmir, Turkey; 4 Katip Çelebi University Faculty of Health Sciences, Department of Public Health Nursing, İzmir, Turkey; 5 Katip Çelebi University Faculty of Medicine, Department of Pediatric Endocrinology, İzmir, Turkey

**Keywords:** Turner syndrome, knowledge levels, questionnaire, education

## Abstract

**Objective:**

Turner syndrome (TS) is one of the most common chromosomal abnormalities and an important cause of short stature and infertility due to ovarian failure in females. The aim was to evaluate the knowledge of TS among physicians and parents of children with TS and to enhance awareness about this subject.

**Methods:**

One hundred and forty physicians were included in the study. The study population comprised 37 pediatricians (26.4%), 15 gynecologists (10.7%), 88 family physicians (62.9%) and 30 parents who had daughters with a diagnosis of TS. Two separate questionnaires were administered to evaluate TS knowledge of physicians and parents.

**Results:**

According to the self-reports of physicians, 49% had insufficient knowledge of TS, while 15.7% indicated that they had no knowledge of TS. The mean percentage of correct answers was 50.71±16.17% for all physicians. When the entire group of physicians was considered, 67.1% of them did not know the approximate incidence of TS, while 14.3% of them incorrectly indicated that TS was a condition that was seen in boys. The mean percentage of correct answers among parents was 68±15%, and there was no difference between the mothers’ and fathers’ correct answer rates (p=0.063). The majority of parents was not aware of TS-associated diseases and increased malignancy risk in TS.

**Conclusion:**

Physician knowledge of TS was poor and that there is a need for continued education about TS at the medical faculty and post-graduate levels.

**What is already known on this topic?**

Turner syndrome (TS) is one of the most commonly observed chromosomal abnormalities, estimated at around 1 in 2500 live births. To the best of our knowledge, there are no studies related to the incidence of TS in Turkey. Nevertheless, in a multicenter study carried out in 2013-2014, 842 patients with TS between 0-18 ages were examined retrospectively in 35 different centers, and the average diagnosis age was determined as 10.2±4.4 years. It is thought that TS is diagnosed at a later age in Turkey.

**What this study adds?**

This study shows that physicians do not have adequate knowledge of TS. Poor knowledge about TS may increase diagnosis delays. The education program about TS should be revised and implemented to address this problem at the medical faculty and post-graduate levels.

## Introduction

Turner syndrome (TS) is a sex chromosome abnormality in females, characterized by partial or complete loss of one of the X chromosomes (1). In nearly half of patients, it can be diagnosed in infancy with the presence of typical clinical findings. While a limited number of patients with TS are diagnosed with short stature during childhood, the rest of them are diagnosed with primary amenorrhea in adolescence (2). Through early diagnosis and appropriate treatment of these patients (growth hormone treatment, estrogen replacement, training and psychological support), they have a chance to participate in academic and social life, and they also achieve nearly normal adult height, bone density and sexual development. Several studies have focused on diagnosing TS earlier (3,4,5). Chronic complications may be prevented by earlier diagnosis and initiating treatment at birth or during infancy. Additionally, parents can deal with the situation more easily with early TS diagnosis (6).

Although TS is common, the exact incidence of TS in Turkey is unknown, and awareness regarding this issue may be inadequate. Patients with TS are diagnosed late in Turkey. Therefore, in this study, we aimed to evaluate the TS knowledge and awareness levels of physicians and parents of children with TS.

## Methods

This descriptive study was a questionnaire survey. The researchers designed two questionnaires; one to be administered to physicians volunteers (n=140) and the other to parents (n=30). The questionnaire for the physicians was developed based on the current literature, guidelines, and expert opinions (7,8). The questionnaire for the parents was developed based on family information flyers from the internet and expert opinions (TS: a guide for families https://turnersyndromefoundation.org/wp-content/uploads/2017/08/New-Turner-Syndrome-Guide-for-Families-Patricia-Reiser-CFNP-and-Marsha-Davenport-MD.pdf and http://nhfv.org/wp-content/uploads/2016/02/Turner-Syndrome-A-Guide-for-Families.pdf). This study included pediatricians, gynecologists, family physicians and parents whose children were diagnosed with TS. Ethics committee approval for the surveys was obtained from the Katip Çelebi University Local Ethics Committee (date: 16 June 2016, ethics approval number: 194). The physicians and parents in question were informed about the questionnaire, and surveys were performed face-to-face by NK after the informed consent form had been signed. An attempt was made to word all questions in a neutral manner. All response data of the participants were analyzed anonymously. No incentives were provided to the respondents.

The first physician survey comprised 18 multiple-choice questions. The first four questions assessed the physicians’ specialties, proficiency regarding TS knowledge, number of years working in their profession and the institutions that they work for. The other 14 questions included TS epidemiology, clinical findings, diagnosis, treatment and follow-up recommendations. There were 19 “yes/no” questions in the survey designed for the parents. The first few questions concerned the demographic features (age, gender and education status) of the parent, and the remaining questions concerned the diagnosis, treatment and follow-up of TS. The patients’ age at diagnosis was obtained from medical records. The answers were evaluated as correct or incorrect by researchers. Examples of the questionnaires applied to both physicians and parents are given in the supplementary documents (Supplementary 1, 2).

### Statistical Analysis

The sample size was calculated according to the estimated size within the sampling universe using the formula referred to as ‘the formula to estimate the number of individuals for a known sample and width of population’ (9).

The analysis of physician survey data was carried out using Statistical Package for the Social Sciences, version 22.0, program (IBM Inc., Armonk, NY, USA), and the percentage distribution was performed using the chi-square test. Descriptive statistics for family surveys, however, were presented as frequency, percentage, average, standard deviation, median, minimum, maximum and range values. In the analysis of differences between measurement values of the two groups, the Mann-Whitney U test or the independent sample t-test were used according to the distribution. A significance test for the difference in two proportions and a Pearson chi-square test were used. A p<0.05 was considered statistically significant.

## Results

A total of 140 physicians working at training and research hospitals, state hospitals, university hospitals and primary care clinics (PCCs) and 30 parents whose children had been diagnosed with TS were included in the study. Of the physicians, 62.9% were family physicians, 26.4% were pediatricians and 10.7% were gynecologists. A total of 50.7% of physicians were working at training and research hospitals, 15% at universities, 3.6% at state hospitals and 30.7% at PCCs and 62.9% of them had been working for 10 years or less.

### Physician Knowledge of Turner Syndrome

Thirty-five percent of physicians self-reported that their knowledge level of TS was adequate, 49.3% indicated that their knowledge was insufficient and 15.7% reported having no knowledge of TS. When all the physicians were considered, the rate of correct answers was 50.71±16.17%. The percentages of correct answers among the 88 family physicians, 37 pediatricians and 15 gynecologists were 46.08±16.51, 56.2±19.02 and 58.3±20.4, respectively.

Responses to several questions related to the frequency and findings of TS are presented in Table 1. For the question concerning chromosomal abnormality, the pediatricians’ accurate answer rate was higher than that of other specialties (p=0.023). The question that referred to the type of hypogonadism was answered incorrectly by 72.1% of all physicians; however, 53.3% of gynecologists answered it correctly.

Gynecologists had the highest accurate answer rate related to fertility and malignancy questions (p=0.028); 64.3% of physicians mistakenly thought that the intelligence level of patients with TS was low. Pediatricians were significantly more well-informed regarding this issue (p=0.018). Approximately 63.6% of the physicians gave incorrect answers to the question regarding estrogen and growth hormone treatment (Table 2).

### Knowledge of Parents About Turner Syndrome

Thirty parents whose children were diagnosed with TS participated in the study. The mean age of girls with TS was 58.8±50.95 months. The median diagnostic age was 66 months (1-168 months). The parents’ percentage of correct answers was 68±15%, and no significant difference was found between mothers and fathers (mothers 74%, fathers 63%; p=0.063). The rate of correct responses among parents was higher than that of physicians, but the difference was not significant. Parent responses to several questions regarding TS are presented in Table 3.

The median correct response rate of primary school graduates was 74% (range, 37-84%), and the median correct response rate of high school or university graduates was 66% (range, 32-89%). There was no significant difference between the parents according to their educational status (p=0.690). However, the median (range) age at diagnosis was significantly younger in children of parents who graduated from high school [21 months (1-120) vs. 90 months (1-168); p=0.008].

## Discussion

TS is one of the most commonly observed chromosomal abnormalities with an incidence of 1 in 2500 live births and effects nearly 1.5 million women in the world (2,10,11). There are no studies related to the incidence of TS in Turkey. Nevertheless, in a multicenter study carried out in 2013-2014, 842 patients with TS between 0-18 ages were examined retrospectively in 35 different centers, and the average diagnosis age was determined as 10.2±4.4 years (12). In a study carried out in England, it was estimated that there were 12,500 TS cases; however, it is known that there are approximately 1000 cases in TS support associations and expert hospital clinics. This means that a large number of cases cannot be diagnosed and do not receive medical care (13). Compared to developed countries, it is thought that TS is diagnosed at a later age in Turkey. This is most likely due to the lower awareness level of Turkish physicians. For this reason, we aimed to investigate TS knowledge and awareness levels of physicians and parents whose children were diagnosed with TS. In the survey, only just over half (50.71±16.17%) of all questions were correctly answered by physicians. It is not possible to compare our results with previous ones because, to the best of our knowledge, no study on this topic has been published in the past in Turkey or in other countries.

The question related to short stature, the most common finding in TS, was not answered properly by 51.1% of family doctors and 29.7% of pediatricians. The growth curve and monitoring of children are important in primary care. The reason for the insufficient knowledge level of physicians may be that they do not encounter such patients because there is no referral chain system. When there is no referral chain, it is difficult for family physicians to maintain health care services, and this situation forms the weakest point of the family medicine practice. In the study by Kringos (14), this situation was given as one of the most important factors why Turkey ranks in the poor category for primary care health services. The final adult height of TS patients is positively related to a younger age at diagnosis and the duration of growth hormone treatment (15). Primary care physicians missing short stature will result in late diagnosis and insufficient benefit from growth hormone treatment for all children who would have benefitted, including girls with TS. When physicians are asked about the intelligence level of children with TS, 64.3% of them answered incorrectly. The inadequate knowledge level of physicians about this issue can cause children diagnosed with TS to be guided in a wrong way and perhaps lead to their exclusion from society. Although patients with TS tend to have some problems with mathematics, these can be overcome with additional time and adequate education. The overall education level of women with TS is equal to or better than that of the overall female population (16). When educational and psychological support is commenced early in TS, it can help academic success and social integration.

The question about high gonadotropin levels in TS was answered more correctly by gynecologists, compared to the physicians in other specialties. This shows that undiagnosed and late-diagnosed girls sought the care of gynecologists with a primary amenorrhea complaint. Although family physicians and pediatricians had inadequate knowledge regarding fertility, 66.7% of gynecologists answered the question correctly. This could be explained by the fact that patients diagnosed with TS consult them for infertility treatment. Most women with TS will be infertile; however, pregnancy has been achieved with oocyte donation and *in vitro* fertilization (17).

Today, many diseases can be diagnosed with simple scanning programs, and in this way, more significant complications can be prevented. The standard approach for cardiac evaluation in TS is echocardiography and four extremity blood pressure measurements that should be performed on every patient at the time of diagnosis (18,19). Even if echocardiography is normal, every patient should be evaluated with magnetic resonance imaging as soon as it is feasible without the need for general anesthesia (7,20). Physicians responded to the question related to cardiovascular disease incorrectly 58.6% of the time. A lack of knowledge can cause late-diagnosed cardiovascular system diseases and increased mortality. There is an increased risk of gonadoblastoma in patients with TS carrying Y chromosome fragmentation, and it is known that remocal of streak gonads are performed by obstetricians and gynecologists. It was not surprising that gynecologists were more knowledgeable than other physicians in terms of the combination of TS and malignancy.

The TS knowledge level of physicians was determined to be unsatisfactory when compared with the knowledge of TS parents. As families research TS in a detailed way after diagnosis, it is not wrong to expect their knowledge level to be higher. Parents want to obtain all information related to the disease since TS is an unknown disease in society and therefore there is an increased level of concern in families. Parents responded with 90% correct answers to the question about TS being a genetic disease. We can imply that this chronic condition led to desperation in families, which increased their solution-oriented quests. Nevertheless, parent knowledge was not sufficient in relation to the diseases accompanying TS and parents should be informed by specialists about this. In a study performed on children with chronic disease, it was reported that there was an important effect of the relationship between the families of hospitalized children and the nurses conducting the care of the sick children. However, the physicians were not well informed about the problems regarding psychosocial adjustment (21,22). Meeting the psychosocial and educational requirements, as well as the medical requirements, of the population affected by chronic disease will increase the childrens’ and families’ quality of life in both the acute and follow-up periods. Additionally, it will positively affect communication between health personnel and families.

The awareness level of primary care family physicians, pediatricians and gynecologists should be enhanced in the areas of early diagnosis and treatment to decrease mortality and morbidity in patients with TS. Our study has revealed troubles related to this issue. It has been shown that major social campaigns are effective in the renewal of knowledge for both families with sick children and physicians, and there is an apparent increase in the early diagnosis of diseases (16). Education programs must be regularly applied to staff by experts in issues such as the requirements of ill children and their parents, and all staff members must be offered counselling services in healthcare organizations. Qualified staff must be employed to apply psychological support, social orientation, and special programs for sick children and their families in healthcare organizations. Therefore, the results show that education programs should be maintained following graduation as well. New early diagnosis strategies should be developed to overcome the delay of treatment in patients with TS.

### Study Limitations

The limitation of this study is the relatively small number of physicians and parents of girls with TS. Accordingly, generalizations from these findings to the total population of physicians and families with children diagnosed with TS must be made cautiously.

## Conclusion

This study indicates that the knowledge about TS of physicians (especially family physicians) was insufficient, although TS is a relatively common disease. To prevent late diagnosis, increased complications and inadequate treatment in patients with TS, post-graduation education programs for physicians should be increased, and the referral chain of the patient must be applied in the health system. The parents’ answers showed that they were worried about TS and the associated problems (short stature, infertility, etc.) and they sought information about TS from clinicians, brochures and the internet. Intermittent information and training programs should be organized for families with TS. Cooperation between the physicians and parents provides better follow-up for these children and better control of the accompanying conditions related to TS.

## Figures and Tables

**Table 1:**
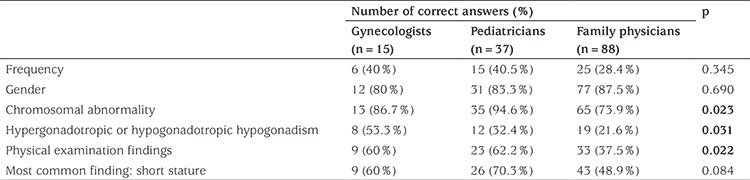
Numbers and percentages of item responses on the survey of physician knowledge about Turner syndrome frequency and findings

**Table 2:**
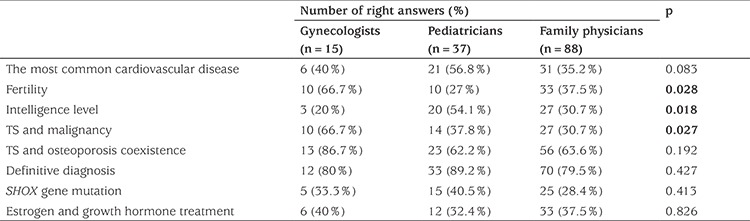
Number and percentages of the item responses on the survey of physician knowledge about diseases associated with Turner syndrome

**Table 3:**
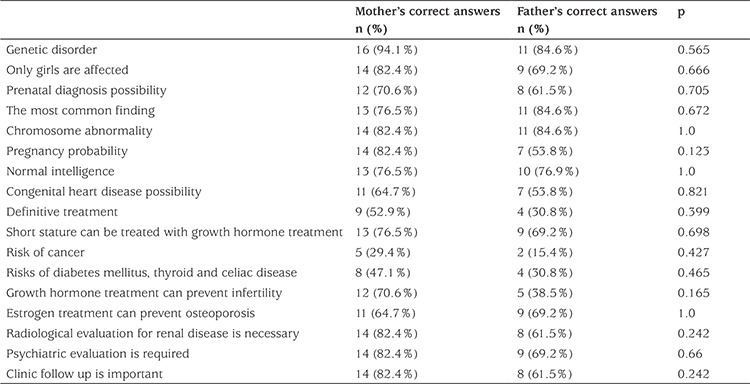
Number and percentages of the item responses on the survey of parent knowledge about Turner syndrome
